# Differential attainment in UK postgraduate medical examinations: examining the relationship between sociodemographic differences and examination performance

**DOI:** 10.1186/s12916-025-04034-w

**Published:** 2025-04-14

**Authors:** Ricky Ellis, Andy Knapton, Jane Cannon, Amanda J. Lee, Jennifer Cleland

**Affiliations:** 1 Institute of Applied Health Sciences, University of Aberdeen, Aberdeen, AB25 2ZD Scotland; 2 General Medical Council, Education & Standards, Regent’s Place, 350 Euston Rd., London, NW1 3AW UK; 3 Medical Statistics Team, Institute of Applied Health Sciences, University of Aberdeen, Aberdeen, AB25 2ZD Scotland; 4 Lee Kong Chian School of Medicine, Nanyang Technological University Singapore, i1 Mandalay Road, Singapore, 308232 Singapore

**Keywords:** Medical education, Assessment, Training

## Abstract

**Background:**

Differential attainment (DA), or differences in performance of groups (rather than individual differences), has been observed in a number of postgraduate medical specialty examinations used in UK medical training. Until now, much of the published research on DA has been limited in scope and size to one specialty, one examination or one type of assessment. This retrospective cohort study addressed this gap by examining the relationship between numerous sociodemographic differences and performance in almost all UK postgraduate medical examinations using a dataset of more than 180,000 examination attempts by UK and international medical graduates, adjusting for prior academic attainment.

**Methods:**

This retrospective cohort study used the UK Medical Education Database (UKMED) to analyse the impact of a range of sociodemographic factors on performance in all UK postgraduate medical examinations aggregated into written and clinical exams. Pass/fail data at the first examination attempt were analysed for all candidates (UK medical school graduates (UKG) and those from non-UK schools (IMG)) sitting an examination between 2014 and 2020. Univariate analyses identified variables to carry forward into multivariate logistic regression models. Informed by previous research, all models were adjusted for prior academic attainment.

**Results:**

180,890 examination first-attempts were made by UKG and IMG candidates, and 121,745 (67.3%) passed at the first attempt. Multivariate regression models showed that place of primary qualification (UKG vs IMG), gender, age, ethnicity, religion, sexual orientation, disability status and working less than full-time were all statistically significant, independent predictors of examination outcomes for all examination candidates. Additionally, there were significant associations between socioeconomic backgrounds and performance for UKGs alone. The strongest independent predictors of failing written and clinical examinations were graduating from a non-UK medical school, having a minority ethnic background and having a registered disability.

**Conclusions:**

This, the largest study of UK postgraduate medical examination outcomes, identified sociodemographic differences that were independently predictive of performance in written and clinical postgraduate medical examinations. Further analysis is now required to ascertain whether these group-level differences exist in each postgraduate medical examination, the majority or a select few.

**Trial registration:**

Not applicable.

**Supplementary Information:**

The online version contains supplementary material available at 10.1186/s12916-025-04034-w.

## Background

Differential attainment (DA), or an awarding gap between groups (rather than individual differences), has been observed across all medical education stages and medical specialties [1], across protected characteristics such as ethnicity [2–16], age [9, 13, 17–22], gender [4, 5, 9, 10, 15, 21–24], and disability/neurodiversity [25–28]. DA is seen between groups that have experienced differences in educational opportunities and socioeconomic backgrounds [9, 29–35], and between UK medical graduates (UKG) and international medical graduates (IMGs) [12, 14, 18, 36–41]. It has been observed across multiple types of assessments, including, but not limited to, outcomes on written and clinical examinations [2–13, 22, 23, 25–28, 35], selection for postgraduate specialty training [14, 19, 30, 41, 42], and appraisal outcomes [17, 21, 35, 43–45]. Such DA indicates the presence of systemic societal and educational biases which hinder individuals’ learning experiences and career progression [2, 46, 47], limit diversity and size of the health workforce [48–50], and ultimately negatively impact patient care [51]. DA is a growing concern for medical educators, policymakers, and the medical community.

In the UK, public authorities such as universities, the National Health Service (NHS) as well as Royal Colleges, the General Medical Council (GMC) and Faculties have a legal duty to address differences between groups with and without certain characteristics protected by the Equality Act 2010 [35, 52, 53]. Understanding patterns of DA in medical training is critical to inform the focus of change efforts and provision of support aiming to reduce these attainment gaps.

However, much of the published research on DA has been limited in scope and size to one specialty, one examination, one type of assessment (e.g. written or clinical) or only including candidates with training numbers, with few exceptions [7, 37]. Similarly, many previous studies have looked only at specific, individual protected characteristics, with ethnicity dominating the research [35, 44, 54, 55]. Focusing on certain characteristics in isolation limits understanding of the factors which are having the greatest impact, and how multiple protected characteristics (e.g. ethnicity and gender) interact in respect of disadvantage [56]. In contrast, the objective of this retrospective cohort study was to examine the relationship between numerous sociodemographic differences and performance in almost all UK postgraduate medical (written and clinical) examinations using a dataset of more than 180,000 examination attempts by UK and international medical graduates.

Note we have used the term “differential attainment” (DA) in this paper rather than “awarding gap”. The latter is becoming more commonplace in medical education. “Awarding gap” explicitly recognises that the issue is not at an individual level, but rather is due to systemic inequalities, and as such it is the responsibility of institutions to address, by ensuring equitable working and learning environments. However, after lengthy consultation with key stakeholders, we adopted the same terminology as that used by the GMC, within the UKMED database and by the wider literature at the time of carrying out the study. This allowed us to contextualise our work and compare it with existing studies. Note, too, that the term “minority group” refers to all groups minoritised within the UK medical environment, whether by underrepresentation, disadvantage, or differences in group-level training and assessment outcomes. This aligns with the definition by Selvarajah et al. [57]; “individuals and populations, including numerical majorities, whose collective cultural, economic, political and social power has been eroded through the targeting of identity in active processes that sustain structures of hegemony.”

## Methods

This retrospective cohort study used the UK Medical Education Database (UKMED) database (https://www.ukmed.ac.uk/). Anonymised data were extracted by the GMC data project manager for all candidates (UK and overseas graduates, candidates with and without a national training number (NTN) at the time of sitting an examination) who attempted UK post-graduate medical examinations between 2014 and 2020 (before the COVID-19 pandemic). Univariate analysis was used to determine the associations between sociodemographic variables available in the UKMED database and first-attempt examination outcomes. Multivariate logistic regression models were then created to identify which variables were independent predictors of success at written and clinical postgraduate medical examinations used in UK medical training.

### Data aggregation

Rules for handling and aggregating data were established before data extraction, and access was granted to the research team. Examination scoring and the score required to pass varies between examinations and sittings. Using a continuous examination outcome variable would considerably limit the interpretation and applicability of the results. Therefore, examination pass/fail was used as the outcome measure. Examination first-attempt results (pass/fail) were used given the strong evidence to show that first-attempt results are the best predictor of later success in medical examinations [58, 59].

All examinations were categorised into either their written components or their Clinical/Objective Structured Clinical Examination (OSCE)/Viva voce (herein described as ‘clinical’ examination) components to create an aggregated comparison between all written versus all clinical postgraduate medical examinations used in the UK. Examinations with fewer than 200 recorded cases were excluded to ensure sufficient statistical power to provide meaningful analyses. These examinations included: Diploma in Pharmaceutical Medicine, Membership of the Faculty of Occupational Medicine, Membership of the Faculty of Sexual and Reproductive Health, Diploma in Otolaryngology – Head and Neck Surgery written, Faculty of Public Health, and several specialty certificate examinations including: Neurology, Infectious Diseases, Medical Oncology and the European Board of Gastroenterology and Hepatology Examination. A detailed description of each examination and its place within medical training pathways lies outside the scope of this paper but can be found online [60]. Additional file 1: Supplementary Table 11 includes the list of all postgraduate examinations included in the final analyses. If a candidate had attempted more than one examination, then their first-attempt results for each examination were included in the analyses.

Self-declared ethnicity and religion were aggregated to align with previous GMC publications and data analyses on differential attainment [46, 61]. This enabled comparison of the current analyses with previous ones. Age was dichotomised into either ≤ 29 years old or > 29 years old at the time of taking the examination. This cut-off is designed to capture those who did a 5–6-year medical degree as an undergraduate and had limited time out of training (e.g. for maternity leave or a “Foundation year 3”) versus more mature candidates who may have taken time out of training, undertaken medicine as a graduate or after several years in a different career before starting medicine. Those with missing data for whether or not they worked less than full-time (LTFT) were assumed to work full-time as the percentage of LTFT candidates in the dataset corresponded with that in recent workforce reports [54]. Data for other demographic variables are presented as held in the UKMED database.

### Measures of socioeconomic status and educational background

Multiple measures of socioeconomic status and educational background are held within UKMED for UKGs only, collected on application to university. Measures of socioeconomic status included Index of Multiple Deprivation (IMD) quintiles and entitlement to income support and free school meals [9, 30, 31, 62]. The second and third of these are self-explanatory but further information is provided within the UKMED data dictionary if required [63]. IMD identifies small zones of deprivation throughout the UK mapped to socioeconomic domains and range from quintile 1 (most deprived) to quintile 5 (least deprived). IMD quintiles were dichotomised into 1 and 2 (as these two quintiles are commonly used in higher education to identify most disadvantaged, or ‘widening participation students’) vs quintiles 3, 4 and 5.

Measures of educational background included the following: high-school type (dichotomised into state (non-fee paying) or fee-paying school), parental education (whether at least one parent is university-educated or not); parental occupation (mapped to national statistics socioeconomic codes on a scale of 1 to 5 and dichotomised into managerial and professional occupations (code 1) vs others (codes 2–5) as used in previous studies) and Participation of Local Areas (POLAR) quintiles [9, 29–31]. The POLAR scoring system classifies areas of the UK according to the level of participation of young people in higher education, which ranges from quintile 1 (lowest participation in higher education) to 5 (highest participation). POLAR scores were dichotomised for analysis, with POLAR quintiles 1 and 2 representing students from the lowest participation areas vs students from quintiles 3, 4 and 5 [9, 30, 31]. Note that both IMD and POLAR scores are based on UK postcodes. Therefore, POLAR and IMD quintiles were included in analyses only for non-graduate entry medical students as, for these groups, POLAR and IMD are most likely to represent the parental/childhood home (rather than a university dwelling).

### Adjustment for prior academic attainment

High school performance has been shown to correlate with success in postgraduate medical examinations [11, 64, 65]. Almost all UK graduates in the UKMED database have linked Universities and Colleges Admissions Service (UCAS) Tariff scores. The UCAS Tariff is a means of allocating points to post-16 qualifications (e.g. A-Levels, Highers, and other high school exit examinations), based on a simple mathematical model which uses a qualification size and grading scale to generate a total number of points. UCAS Tariff scores are thus a surrogate measure of ‘prior academic attainment’. Information regarding how UCAS Tariff scores are calculated can be found at https://www.ucas.com/.

International medical graduates (IMGs) who’s place of primary medical qualification (PMQ) is outside of the UK do not have a UCAS tariff. Thus, outcome on the Professional and Linguistic Assessments Board (PLAB), a test required to register for a GMC license to practice medicine, was used as a measure of prior attainment for IMGs. The PLAB aims to ‘check that IMGs know and can do the same as a doctor starting the second year of their Foundation Programme training in the UK’ [66]. The PLAB is known to show predictive validity and correlates with later performance on postgraduate examinations [37, 45, 67].

Thus, individual-linked UCAS Tariff scores (for UKGs) and PLAB scores (for IMGs) relative to the pass mark were each converted to continuous *z*-scores to take account of changes to pass marks between each examination diet within the study period. While not perfect measures (i.e. both could potentially exhibit group-level attainment differences and UCAS tariffs are more historical scores compared to the PLAB test which is taken more recently in trainees’ careers) these scores provided a numerical measure of prior academic attainment for each candidate within the dataset.

### Statistical analysis

Univariate analysis was used to determine the associations with first-attempt examination outcomes. To avoid a high level of multi-collinearity within the MV regression models, Spearman’s Rho correlation coefficients were first calculated for each measure of socioeconomic status and educational background (Additional file 1: Supplementary Table 2). Where a high correlation coefficient was found between two variables representing either socioeconomic status or educational background, only one was entered into regression models. The following measures were therefore not carried forward into regression models: eligibility for free school meals and income support (note: IMD variable was retained, which captures measures of childhood socioeconomic status) and POLAR scores (note: educational opportunity is captured within the retained variables IMD and school type). Missing data (including where data was not declared by individuals during data collection exercises) were excluded from regression analyses (all analyses were therefore performed on a complete-case basis), and the total cohort used in each analysis (*n*) is stated in each table.

Logistic regression (LR) models were created using backwards conditional MV regression analyses. Two LR models were created, both adjusted for measures of prior academic attainment. The first included all candidates (all UK graduates (UKG) and international medical graduates (IMGs)), the second LR model included only UKGs as more granular sociodemographic data were available for this group. For example, less than 5% of IMGs had matched data for socioeconomic status, education background or first language. Likewise, 80% of UKGs had missing First language data, preventing this variable from being included in LR models. Only variables that remained significant in the final MV model after adjusting for all other variables are presented. All analyses were conducted using SPSS® for Windows v24.0 (IBM Corp, Armonk, NY, USA). In line with the Higher Education Statistics Agency data standards (www.hesa.ac.uk), all counts presented have been rounded to the nearest 5 to ensure person-level anonymity [68].

### Ethics

No formal ethical approval was required for this study of existing UKMED data. UKMED has received ethics exemption for projects using exclusively UKMED data from Queen Marys University of London Ethics of Research Committee on behalf of all UK medical schools (https://www.ukmed.ac.uk/documents/UKMED_research_projects_ethics_exemption.pdf). The Intercollegiate Committee for Basic Surgical Examinations (ICBSE) and its Internal Quality Assurance Subcommittee, which monitors MRCS standards, research and quality, approved this study.

### Patient and public involvement

Patients and the public were not involved in the design, conduct or reporting of this study.

## Results

Between 2014 and 2020, 180,890 first-attempts were made at UK postgraduate medical examinations by UKG or IMG candidates. A total of 121,745 (67.3%) passed at the first attempt. Excluding candidates with missing data, the largest groups within each sociodemographic factor were UKG (73.2%), Female (51.2%), age > 29 years old (53.7%), White (54.3%), no religion (35.6%), heterosexual/ straight (96.1%), no disability (94.3%) and not LTFT (92.9%; less than full-time).

Of UK graduates, 75.4% (*n* = 99,840) passed at the first attempt. Excluding candidates with missing data, the largest groups within each sociodemographic factor for UKGs only were Female (54.5%), age ≤ 29 years old (56.8%), White (64.6%), no religion (46.4%), heterosexual/ straight (95.3%), no disability (93.7%), not LTFT (92.7%), English as first language (78.9%), university educated parents (68.8%), parents in managerial or professional occupations (87.5%), POLAR quintiles III-V (86.7%), IMD quintiles III-IV (80.8%), attended state/non-fee paying schools (70.1%), not eligible for income support (86.4%) and not eligible for free school meals (91.5%).

Of international graduates, 45.2% (*n* = 21,905) passed at the first attempt. Excluding candidates with missing data, the largest groups within each sociodemographic factor for IMGs only were Male (57.9%), age > 29 years old (82.4%), Asian or Asian British (52.6%), Muslim (33.2%), heterosexual/ straight (81.1%), no disability (96.8%) and not LTFT (93.4%).

### UK graduate and international medical graduate combined results

Univariate analysis of postgraduate medical examination first attempt pass rates by sociodemographic variables for UKGs and IMGs is shown in Table 1. The logistic regression (LR) model heatmap showing predictors of success and failure at the first attempt at all postgraduate written and all postgraduate clinical examinations for UKGs and IMGs combined after accounting for prior academic performance is shown in Table 2. The numerical logistic regression results containing odds ratios (OR) and 95% confidence intervals (CI) can be found in Table 3. In total, 69,595 first-attempts at written examinations had matched data and were included in the LR analysis, and 38,485 first-attempts at clinical examinations had matched data and were included.

**Table 1:** Univariate analysis of postgraduate medical examination first attempt pass rates by sociodemographic variables for UK (UKG) and international medical graduates (IMG). Values presented as percentage pass rate and (number that passed/total number of first attempts (*n*))

	Percentage pass rate at first attempt (number passed/total number of first attempts)		
	UK and international medical graduates	UK medical graduates	International medical graduates
***N* in cohort**	180,890	132,370	48,520
**PMQ,** *P*-value	**< 0.001**	N/A	N/A
UK	75.4% (99,840/132,370)	75.4% (99,840/132,370)	N/A
IMG	45.2% (21,905/48,515)	N/A	45.2% (21,905/48,515)
Missing (*N*)	0	0	0
**Gender,** *P*-value	**< 0.001**	** < 0.001**	**< 0.001**
Males	65.0% (57,415/88,280)	74.7% (44,970/60,165)	44.3% (12,450/28,115)
Females	69.5% (64,330/92,605)	76.0% (54,870/72,205)	46.4% (9460/20,400)
Missing (*N*)	0	0	0
**Age,** *P*-value	**< 0.001**	**< 0.001**	**< 0.001**
≤ 29 years	71.9% (60,260/83,780)	74.6% (56,140/75,220)	48.2% (4120/8555)
> 29 years	63.3% (61,485/97,110)	76.4% (43,700/57,150)	44.5% (17,785/39,960)
Missing (*N*)	0	0	0
**Ethnicity,** *P*-value	**< 0.001**	**< 0.001**	**< 0.001**
White	77.0% (72,550/94,180)	79.5% (68,055/85,565)	52.2% (4495/8615)
Asian or Asian British	56.7% (31,490/55,530)	67.6% (20,275/29,995)	43.9% (11,215/25,535)
Black or Black British	46.9% (4340/9250)	60.1% (1790/2980)	40.7% (2550/6270)
Mixed	68.2% (4055/5945)	74.2% (3575/4815)	42.5% (480/1130)
Other Ethnic Groups	55.3% (4755/8600)	65.1% (2655/4075)	46.4% (2100/4525)
Missing (*N*)	7385	4940	2445
**Religion,** *P*-value	**< 0.001**	**< 0.001**	**< 0.001**
No religion	77.5% (40,235/51,895)	79.0% (38,485/48,740)	55.5% (1750/3155)
Buddhist	57.9% (2145/3700)	64.7% (1235/1910)	50.7% (905/1785)
Christian	68.4% (32,015/46,805)	75.7% (27,075/35,760)	44.7% (4940/11,045)
Hindu	56.5% (7995/14,160)	69.2% (4370/6310)	46.2% (3625/7850)
Jewish	74.1% (725/975)	76.0% (675/890)	54.1% (45/85)
Muslim	50.5% (12,375/24,525)	64.6% (5425/8395)	43.1% (6950/16,130)
Sikh	64.9% (1305/2015)	70.1% (1145/1630)	42.7% (165/380)
Other	63.2% (1150/1820)	69.1% (985/1425)	41.8% (165/395)
Missing (*N*)	35,000	27,310	7690
**Sexual orientation,** *P*-value	**< 0.001**	**0.001**	**0.001**
Heterosexual/straight	67.0% (92,265/137,735)	75.6% (74,410/98,405)	45.4% (17,855/39,335)
Bisexual	67.5% (880/1305)	69.2% (770/1045)	41.3% (105/260)
Lesbian/gay/homosexual	72.2% (2755/3815)	74.0% (2550/3440)	55.1% (205/370)
Other	65.9% (330/500)	68.9% (285/410)	52.3% (45/90)
Missing (*N*)	37,535	29,070	8465
**Disability,** *P*-value	**< 0.001**	**< 0.001**	**< 0.001**
No vs	70.3% (99,475/141,485)	76.4% (85,395/111,715)	47.3% (14,080/29,770)
Yes	62.0% (5270/8500)	65.5% (4925/7520)	35.2% (345/980)
Missing (*N*)	30,905	13,135	17,765
**LTFT,** *P*-value	**< 0.001**	**< 0.001**	**< 0.001**
No	66.9% (112,515/168,060)	75.0% (92,075/122,740)	45.1% (20,440/45,325)
Yes	72.0% (9230/12,830)	80.6% (7765/9635)	45.9% (1465/3195)
Missing (*N*)	0	0	0
**Prior attainment (*z* score),** *P*-value	** < 0.001**	**< 0.001**	**< 0.001**
Pass mean	0.1210	0.0656	0.5599
Fail mean	−0.0542	−0.1194	0.0674
Missing (*N*)	22,500	3245	19,260

**Table 2:**
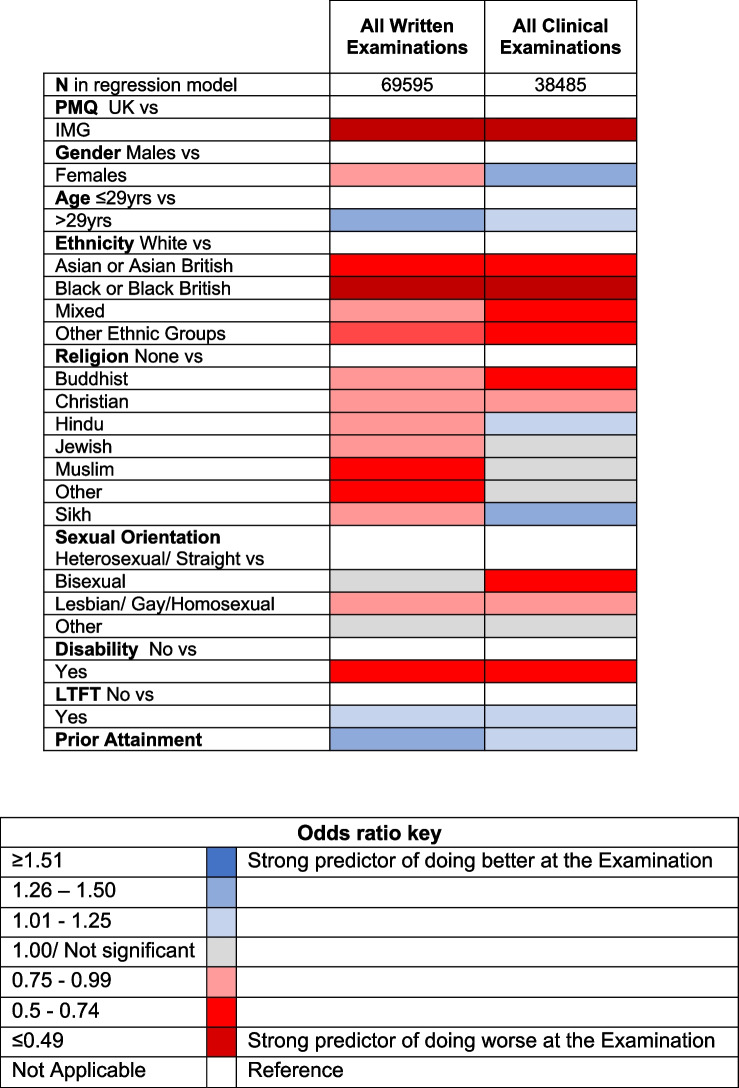
Logistic regression model heatmap (and odds ratio key) showing predictors of success at the first attempt at all combined written and all combined clinical postgraduate medical examinations for UK (UKG) and international medical graduates (IMG) after accounting for prior academic performance. The first category displayed within each variable was used as the reference

**Table 3:** Logistic regression model showing predictors of success at the first attempt at all combined written and all combined clinical postgraduate medical examinations for UK (UKG) and International Medical graduates (IMG) after accounting for prior academic performance. The first category displayed within each variable was used as the reference. Results are presented as odds ratio (95% confidence interval) and statistically significant effect sizes are boldened. Examination abbreviations are outlined in the examination key

	All written examinations	All clinical examinations
***N* in analysis**	69,595	38,485
**PMQ,** UK vs		
IMG	**0.35**	**0.25**
	**(0.33–0.37)**	**(0.23–0.27)**
**Gender,** Males vs		
Females	**0.88**	**1.33**
	**(0.85–0.91)**	**(1.26–1.40)**
**Age,** ≤ 29 years vs		
> 29 years	**1.32**	**1.14**
	**(1.27–1.37)**	**(1.08–1.20)**
**Ethnicity,** White vs		
Asian or Asian British	**0.59**	**0.50**
	**(0.56–0.62)**	**(0.46–0.54)**
Black or Black British	**0.49**	**0.49**
	**(0.45–0.53)**	**(0.43–0.54)**
Mixed	**0.78**	**0.63**
	**(0.71–0.86)**	**(0.55–0.72)**
Other Ethnic Groups	**0.62**	**0.52**
	**(0.57–0.68)**	**(0.46–0.59)**
**Religion,** None vs		
Buddhist	**0.79**	**0.68**
	**(0.71–0.89)**	**(0.57–0.80)**
Christian	**0.77**	**0.93**
	**(0.74–0.80)**	**(0.87–0.99)**
Hindu	**0.81**	**1.12**
	**(0.75–0.87)**	**(1.00–1.25)**
Jewish	**0.78**	0.81
	**(0.64–0.96)**	(0.60–1.10)
Muslim	**0.67**	0.97
	**(0.63–0.72)**	(0.88–1.06)
Other	**0.66**	0.88
	**(0.57–0.77)**	(0.70–1.11)
Sikh	**0.84**	**1.28**
	**(0.73–0.96)**	**(1.05–1.56)**
**Sexual orientation,** Heterosexual/straight vs		
Bisexual	0.86	**0.74**
	(0.73–1.02)	**(0.55–0.98)**
Lesbian/gay/homosexual	**0.82**	**0.81**
	**(0.74–0.91)**	**(0.70–0.94)**
Other	0.77	0.91
	(0.57–1.06)	(0.57–1.46)
**Disability,** No vs		
Yes	**0.55**	**0.66**
	**(0.51–0.58)**	**(0.60–0.73)**
**LTFT,** No vs		
Yes	**1.24**	**1.14**
	**(1.15–1.33)**	**(1.05–1.25)**
**Prior attainment**	**1.39**	**1.18**
	**(1.37–1.42)**	**(1.14–1.21)**

Place of primary medical qualification (PMQ; UK or overseas) was the strongest predictor of failure at UK postgraduate medical examinations, regardless of examination format (written or clinical). IMGs were 65% less likely to pass a written examination (OR 0.35 (95% CI 0.33 to 0.37)) and 75% less likely to pass a clinical examination (OR 0.25 (95% CI 0.23 to 0.27)) at the first attempt compared to UKGs after adjusting for other sociodemographic factors and prior academic attainment.

Different patterns of attainment were seen according to gender and depending on examination format. Female candidates were significantly less likely to pass written examinations (OR 0.88 (95% CI 0.85 to 0.91)) but significantly more likely to pass clinical examinations (OR 1.33 (95% CI 1.26 to 1.40)) at the first attempt compared to male candidates. Older candidates (> 29 years of age) were significantly more likely to pass written (OR 1.32 (95% CI 1.27 to 1.37)) and clinical (OR 1.14 (95% CI 1.08 to 1.20)) examinations at the first attempt compared to younger candidates.

After adjusting for other sociodemographic factors and prior academic attainment, ethnicity was a strong predictor of examination outcomes. Minority ethnic groups were significantly less likely to pass written and clinical examinations at the first attempt compared to White candidates. The biggest attainment gap existed for candidates identifying as Black or Black British, who were less than half as likely to pass written (OR 0.49 (95% CI 0.45 to 0.53)) and clinical (OR 0.49 (95% CI 0.43 to 0.54)) examinations compared to White candidates.

A strong correlation (Spearman’s Rho) existed between ethnicity and religion *r* = 0.506 (*p* < 0.001) which is shown in Additional file 1: Supplementary Table 3. Of those who stated a religion, even after adjusting for other sociodemographic variables including ethnicity and prior attainment, there was DA according to religious beliefs. Candidates with religious beliefs were significantly less likely to pass written examinations at the first attempt compared to their peers who did not identify as having a religion. Attainment patterns differed considerably for clinical examinations. Candidates identifying as Buddhist and Christian were significantly less likely to pass clinical examinations at the first attempt (OR 0.68 (95% CI 0.57 to 0.80) and OR 0.93 (95% CI 0.87 to 0.99), respectively). On the other hand, candidates identifying as Hindu and Sikh were significantly more likely to pass (OR 1.12 (95% CI 1.00 to 1.25) and OR 1.28 (95% CI 1.05 to 1.56), respectively).

Significant differences in attainment were found according to sexual orientation. Lesbian, gay or homosexual candidates were nearly 20% less likely than straight or heterosexual candidates to pass written and clinical examinations at the first attempt (OR 0.82 (95% CI 0.74 to 0.91) and OR 0.81 (95% CI 0.70 to 0.94) respectively). Bisexual candidates were 26% less likely to pass clinical examinations (OR 0.74 (95% CI 0.55 to 0.98)). Identifying as bisexual was not found to be an independent statistically significant predictor of written examination outcomes.

Disability status was a strong predictor of examination outcomes. Candidates with registered disabilities were 45% less likely to pass written examinations and 34% less likely to pass clinical examinations than their peers without disabilities (OR 0.55 (95% CI 0.51 to 0.58) and OR 0.66 (95% CI 0.60 to 0.73) respectively). LTFT appeared to be a protective factor with LTFT candidates being 24% more likely to pass written and 14% more likely to pass clinical examinations (OR 1.24 (95% CI 1.15 to 1.33) and OR 1.14 (95% CI 1.05 to 1.25), respectively).

As per previous studies, prior academic attainment (individual performance on the PLAB or UCAS tariff) remained a predictor of future success at medical written and clinical examinations (OR 1.39 (95% CI 1.37 to 1.42) and OR 1.18 (95% CI 1.14 to 1.21), respectively).

### UK graduate only results

The results of univariate analyses between sociodemographic variables and pass rates at all UK postgraduate medical examinations split by written vs clinical components are shown in Table 4. The logistic regression model heatmap showing predictors of success and failure at the first attempt at all postgraduate written and clinical examinations for UKG (after accounting for prior academic performance) is shown in Table 5. The numerical logistic regression results containing odds ratios and 95% confidence intervals can be found in Table 6. In total, 48,430 first attempts at written examinations had matched data and were included in the LR analysis, and 27,380 first attempts at clinical examinations had matched data and were included.

**Table 4:** Univariate analysis using chi-squared testing of all combined written and all combined clinical postgraduate medical examination first attempt pass rates by sociodemographic variables for UK graduates. Values presented as percentage pass rate and (number that passed/total cohort number (*n*))

	All written examinations	All clinical examinations
***N* in cohort**	**83,400**	**48,295**
**Gender,** *P*-value	**< 0.001**	**< 0.001**
Males	73.8% (27,805/37,675)	76.2% (16,915/22,190)
Females	72.3% (33,080/45,720)	82.3% (21,480/26,105)
Missing (*N*)	**0**	**0**
**Age,** *P*-value	0.001	0.132
≤ 29 years	72.6% (37,810/52,075)	79.2% (18,305/23,110)
> 29 years	73.7% (23,075/31,325)	79.8% (200,090/25,190)
Missing (*N*)	0	0
**Ethnicity,** *P*-value	**< 0.001**	**< 0.001**
White	77.3% (41,440/53,635)	83.3% (26,245/31,490)
Asian or Asian British	64.9% (12,335/19,010)	72.2% (7840/10,850)
Black or Black British	55.6% (1065/1915)	68.0% (715/1050)
Mixed	72.8% (2245/3080)	76.6% (1305/1705)
Other Ethnic Groups	62.4% (1645/2635)	69.8% (990/1415)
Missing (*N*)	3125	1785
**Religion,** *P*-value	**< 0.001**	**< 0.001**
No religion	77.5% (24,280/31,330)	81.6% (14,000/17,160)
Buddhist	63.8% (820/1280)	66.5% (415/625)
Christian	72.5% (16,200/22,355)	81.0% (10,715/13,220)
Hindu	65.4% (2595/3965)	75.6% (1750/2315)
Jewish	74.3% (435/585)	79.4% (235/300)
Muslim	60.5% (3225/5330)	71.9% (2185/3040)
Sikh	65.6% (660/1010)	77.4% (475/615)
Other	65.8% (610/925)	74.6% (365/490)
Missing (*N*)	16,625	10,531
**Sexual orientation,** *P*-value	0.247	** < 0.001**
Heterosexual/straight	73.1% (45,530/62,325)	80.0% (28,490/35,610)
Bisexual	71.9% (540/750)	78.6% (230/290)
Lesbian/gay/homosexual	73.1% (1660/2270)	76.0% (875/1150)
Other	67.8% (180/265)	70.8% (100/145)
Missing (*N*)	17,795	11,100
**Disability,** *P*-value	**< 0.001**	**< 0.001**
No vs	74.1% (52,000/70,135)	80.3% (32,960/41,055)
Yes	61.3% (3075/5020)	73.8% (1810/2455)
Missing (*N*)	8245	4790
**LTFT,** *P*-value	**< 0.001**	**< 0.001**
No	72.8% (57,100/78460)	79.0% (34,485/43680)
Yes	76.7% (3785/4935)	84.7% (3910/4620)
Missing (*N*)	0	0
**English first language,** *P*-value	**< 0.001**	**< 0.001**
Yes	72.6% (12,030/16,570)	79.7% (3465/4345)
No	62.4% (2725/4370)	69.9% (855/1225)
Missing (*N*)	62,460	42,730
**Parental degree,** *P*-value	**< 0.001**	0.332
Yes	76.5% (26,055/34,050)	81.0% (18,780/23,190)
No	72.5% (11,095/15,300)	80.5% (8590/10665)
Missing (*N*)	34,050	14,440
**Parental occupation,** *P*-value	**< 0.001**	**0.005**
Managerial/professional	72.0% (23,295/32,345)	79.1% (9605/12145)
Other occupations	67.9% (3090/4555)	76.1% (1360/1785)
Missing (*N*)	46,495	34,365
**POLAR quintile,** *P*-value	**< 0.001**	**< 0.001**
III–V other neighbourhood	75.0% (45,170/60,195)	81.2% (29,330/36,135)
I–II low participation neighbourhood	71.5% (6575/9205)	78.9% (4440/5625)
Missing (*N*)	13,995	6535
**IMD quintile,** *P*-value	**< 0.001**	**< 0.001**
III–IV (least deprived)	75.6% (44,785/59,210)	81.7% (28,240/34,565)
I–II (most deprived)	67.8% (9540/14,070)	77.2% (6355/8230)
Missing (*N*)	10,120	5500
**School type,** *P*-value	**< 0.001**	0.948
State	73.8% (35,715/48,380)	80.8% (22,645/28,040)
Fee-paying	75.4% (15,525/20,575)	80.7% (9730/12050)
Missing (*N*)	14,440	8205
**Income support,** *P*-value	**< 0.001**	**< 0.001**
No	76.2% (30,085/39,485)	81.6% (22,300/27,320)
Yes	72.0% (4570/6350)	78.3% (3240/4135)
Missing (*N*)	37,565	16,845
**Free school meals,** *P*-value	**< 0.001**	**< 0.001**
No	76.1% (33,240/43,680)	81.4% (24,400/29,960)
Yes	69.0% (2815/4075)	76.4% (2115/2770)
Missing (*N*)	35,640	15,565
**Prior attainment (*z* score),** *P*-value	**< 0.001**	**< 0.001**
Pass mean	0.0802	0.0445
Fail mean	− 0.1440	− 0.0630
Missing (*N*)	1765	1300

**Table 5:**
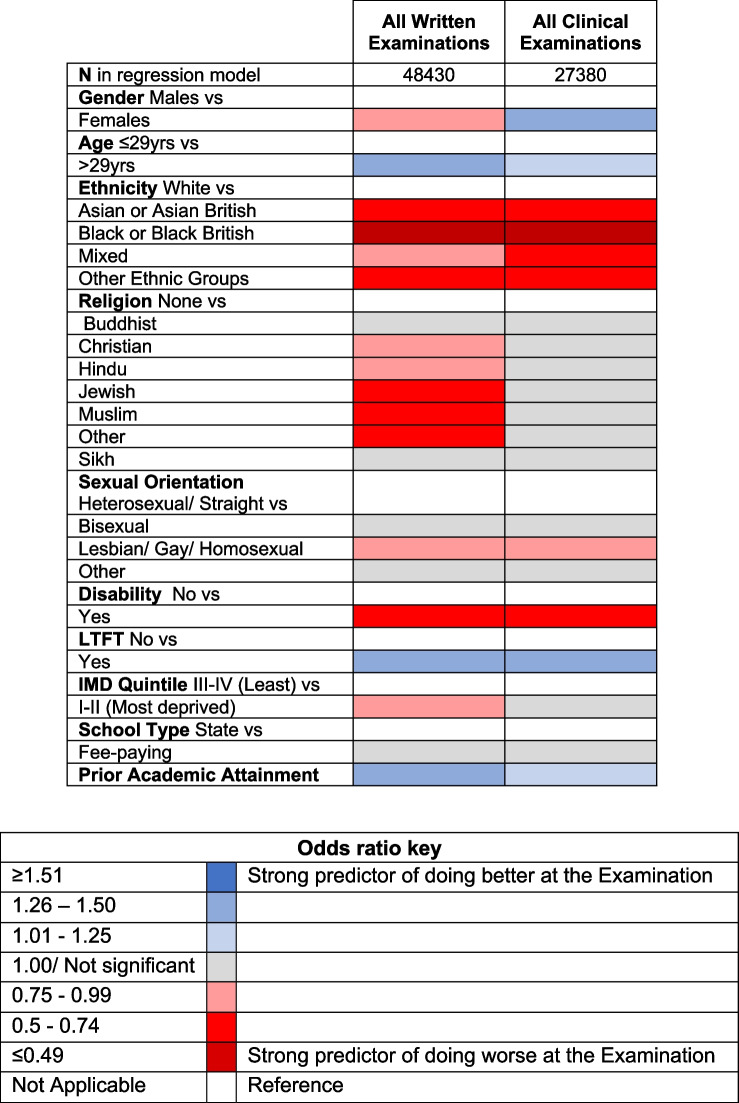
Logistic regression model heatmap (and odds ratio key) showing predictors of success at the first attempt at all combined written and all combined clinical postgraduate medical examinations for UK medical school graduates (UKG) after accounting for prior academic performance. The first category displayed within each variable was used as the reference

**Table 6:** Logistic regression model showing predictors of success at the first attempt at all combined written and all combined clinical postgraduate medical examinations for UK medical school graduates (UKG) after accounting for prior academic performance. The first category displayed within each variable was used as the reference. Results are presented as odds ratio (95% confidence interval) and statistically significant effect sizes are boldened. ‘x’ denotes an invalid model due to small cohort sizes and ‘-’ denotes where a variable has not met statistical significance within the final model

	All written examinations	All clinical examinations
**N in analysis**	48,430	27,380
**Gender,** Males vs		
Females	**0.89**	**1.33**
	**(0.85–0.93)**	**(1.25–1.42)**
**Age,** ≤ 29 years vs		
> 29 years	**1.32**	**1.15**
	**(1.26–1.39)**	**(1.08–1.23)**
**Ethnicity,** White vs		
Asian or Asian British	**0.62**	**0.60**
	**(0.58–0.67)**	**(0.56–0.64)**
Black or Black British	**0.44**	**0.42**
	**(0.39–0.50)**	**(0.35–0.51)**
Mixed	**0.84**	**0.66**
	**(0.75–0.93)**	**(0.57–0.77)**
Other Ethnic Groups	**0.61**	**0.55**
	**(0.53–0.70)**	**(0.46–0.67)**
**Religion,** None vs		
Buddhist	0.83	-
	(0.66–1.04)	-
Christian	**0.79**	-
	**(0.75–0.82)**	-
Hindu	**0.82**	-
	**(0.74–0.92)**	-
Jewish	**0.71**	-
	**(0.57–0.88)**	-
Muslim	**0.69**	-
	**(0.63–0.76)**	-
Other	**0.68**	-
	**(0.57–0.81)**	-
Sikh	0.88	-
	(0.74–1.04)	-
**Sexual orientation,** Heterosexual/straight vs		
Bisexual	0.86	0.79
	(0.71–1.04)	(0.56–1.12)
Lesbian/gay/homosexual	**0.80**	**0.80**
	**(0.71–0.90)**	**(0.68–0.95)**
Other	0.83	0.91
	(0.57–1.20)	(0.50–1.65)
**Disability,** No vs		
Yes	**0.53**	**0.65**
	**(0.49–0.57)**	**(0.58–0.74)**
**LTFT,** No vs		
Yes	**1.30**	**1.33**
	**(1.18–1.44)**	**(1.17–1.51)**
**IMD quintile,** III–IV (least) vs		
I–II (Most deprived)	**0.84**	-
	**(0.79–0.89)**	-
**School type,** State vs		
Fee-paying	-	-
	-	-
**Prior academic attainment**	**1.35**	**1.12**
	**(1.31–1.38)**	**(1.08–1.16)**

Attainment differences according to gender remained the same as that seen in the high-level all-candidates analyses (of UKG and IMG combined), with females being significantly less likely to pass written examinations (OR 0.89 (95% CI 0.85 to 0.93)) but significantly more likely to pass clinical examinations (OR 1.33 (95% CI 1.25 to 1.42)) at the first attempt compared to males. Also similar was the finding that older candidates (> 29 years of age) were significantly more likely to pass written (OR 1.32 (95% CI 1.26 to 1.39)) and clinical (OR 1.15 (95% CI 1.08 to 1.23)) examinations at the first attempt compared to younger candidates.

Ethnicity remained a strong predictor of examination outcomes amongst UKGs after adjusting for other sociodemographic factors and prior academic attainment. UKGs from minority ethnic groups were significantly less likely to pass both written and clinical examinations at the first attempt compared to White candidates. Similar to the all-candidates analyses, the biggest attainment gap was between White candidates and Black or Black British candidates, who were 56% less likely to pass written (OR 0.44 (95% CI 0.39 to 0.50)) and 58% less likely to pass clinical (OR 0.42 (95% CI 0.35 to 0.51)) examinations compared to White candidates.

A moderate correlation (Spearman’s Rho) was found between ethnicity and religion *r* = 0.396 (*p* < 0.001), which is shown in Additional file 1: Supplementary Table 4. Candidates who identified as Christian, Hindu, Jewish, Muslim or Other religions were significantly less likely to pass written examinations at the first attempt compared to their peers who did not identify as having a religion. No statistically significant difference in attainment was found between Buddhist and Sikh candidates vs candidates with no religion for written examinations. In contrast to the high-level all-candidates analysis, religion was not a statistically significant predictor of clinical examination outcomes for UKGs after adjusting for other sociodemographic factors and prior academic attainment.

Similar to the high-level all-candidates analyses, significant differences in attainment were found according to sexual orientation amongst UKGs. Lesbian, gay or homosexual candidates were 20% less likely than straight or heterosexual candidates to pass written and clinical examinations at the first attempt (OR 0.80 (95% CI 0.71 to 0.90) and OR 0.80 (95% CI 0.68 to 0.95), respectively). A notable difference to the high-level all-candidates analysis, was that identifying as bisexual was not found to be an independent statistically significant predictor of clinical examination outcomes for UKGs, indicating that the attainment differences found for this group in the high-level all-candidates analysis were largely experienced by IMGs.

Disability status remained a strong predictor of written and clinical examination outcomes. Candidates with registered disabilities were 47% less likely to pass written examinations and 35% less likely to pass clinical examinations than their peers without disabilities (OR 0.53 (95% CI 0.49 to 0.57) and OR 0.65 (95% CI 0.58 to 0.74), respectively). LTFT remained a protective factor, with LTFT UKGs being significantly more likely to pass written and clinical examinations (OR 1.30 (95% CI 1.18 to 1.44) and OR 1.33 (95% CI 1.17 to 1.51), respectively).

UKGs from more socioeconomically deprived backgrounds (IMD quintiles I–II) were 16% less likely to pass written examinations at the first attempt compared to their peers from less deprived backgrounds (OR 0.84 (95% CI 0.79 to 0.89)). IMD quintile was not found to be a statistically significant predictor of clinical examination outcomes. School type was not found to be a statistically significant predictor of either written or clinical examination outcomes. Similar to the high-level all-candidates analyses, prior academic attainment (in this case, UCAS tariff scores) remained a predictor of future success at medical written and clinical examinations (OR 1.35 (95% CI 1.31 to 1.38) and OR 1.12 (95% CI 1.08 to 1.16) respectively).

## Discussion

In this study we set out to examine the relationship between numerous sociodemographic differences and performance in almost all UK postgraduate medical (written and clinical) examinations. Using a dataset of more than 180,000 examination attempts by UK (UKGs) and international medical graduates (IMGs), we identified that, after accounting for prior academic attainment, differences in performance were found according to place of primary qualification, gender, age, ethnicity, religion, sexual orientation, disability and LTFT status across all UK postgraduate examination candidates (UKGs and IMGs). Additionally, there were significant associations between socioeconomic backgrounds and performance for UKGs. Place of primary medical qualification (PMQ; UK or overseas) was the strongest predictor of outcomes, and the strongest independent predictors of failing written and clinical examinations were place of primary medical qualification, ethnicity and disability status.

We considered a greater number of potentially influencing sociodemographic variables than previous similar studies of group differences [2–13, 22, 23, 25–28, 35], including many individual differences that have been historically neglected in DA studies (e.g. religion, sexual orientation, disability, LTFT). Doing so highlighted to us that different variables, or social positions [69], are not independent of each other. Instead, they intersect at the individual level (e.g. ethnicity, gender and sexual orientation). In other words, it is no longer adequate to look at sociodemographic variables in isolation or semi-isolation (e.g. gender, ethnicity) in studies examining the attainment gap. Instead, we need to look at unique experiences with consideration to the intersectionality of groups. By doing so, we may gain new insights, including identifying what groups are more disadvantaged than others, and thus be able to target interventions more effectively. This notion of intersectionality has long been used in qualitative studies of identity and marginalisation, but it is now gaining traction in quantitative research across disciplines, including epidemiology and public health [70, 71].

### Comparison with previous literature

Our findings that place of primary medical qualification was a very strong predictor of failing aligns with previous literature on this topic [12, 18, 36–39]. Language issues and other biases related to examination content and format may contribute, at least in part, to this finding [37]. However, a recent scoping review suggests that IMGs are subject to numerous common inequitable workplace experiences and that these experiences are important in career progression [72]. Given IMGs make up a large proportion of the medical workforce in many countries, including the UK, it is critical to better explore and address needs and challenges faced by IMGs in the UK and indeed across the world.

We found that age was a predictor of performance. This may be at least in part explained by the fact that older candidates might be doing different examinations: some postgraduate examinations are taken later or earlier in the training pathway (Membership versus Fellowship examinations in certain specialties). However, previous research has highlighted that, in the same examination, older candidates tend to do less well than their younger peers [9, 13, 18, 22]. The data do not allow us to examine the reasons for this. It may be that older candidates have other commitments which impact examination revision (e.g. parental or caring responsibilities), or have had progression delays earlier in the training pipeline. A within-subjects longitudinal quantitative study is needed to examine differing progression through training and whether the awarding gap narrows or widens over time.

That our findings were similar for written and clinical examinations suggests that examiner bias is not a major factor in group-level differences [2, 73]. However, those in charge of examinations need to ensure that their processes and training are fit for purpose and equitable.

### Strengths and limitations

Big data studies such as this are inevitably limited by the data that are available. While the UKMED database is one of the world’s most complete and comprehensive medical education databases, it does not capture other factors that may impact performance on examinations, such as place of training, training opportunities, access to revision resources, and study practices. There is some evidence that the first of these, place of training, is associated with performance in UK medical students and doctors in training [74, 75]. However, these studies also show that it is not the place itself which is important, but that high-status medical schools and training providers attract stronger candidates. What is clear is that there is a complex relationship between sociodemographic characteristics, assessment performance and opportunities as learners progress through medical education and training.

There is also a high degree of missing data for some important variables (e.g. first language), which prevented their inclusion in analyses. Additionally, despite the considerable size of the study population, analysing such a large number of sociodemographic differences for granularity reduces the size of the cohorts for MV analyses, especially when some variables have a higher proportion of missing data (e.g. religion, sexual orientation and disability). This issue has the potential to impact the statistical power and generalisability of some results.

Throughout the study, variables were often dichotomised or categorised (see the “ Methods” section). This approach is pragmatic and commonly used when studying group differences [35], but fails to fully acknowledge the intersectionality of identities [56, 69] and heterogeneity within groups. For example, disabilities vary considerably in severity, type, and impact on activities of daily living and workplace experiences, but these differences are hidden by data aggregation [54, 76]. Similarly, IMGs move to the UK from all over the world [54]. IMGs and UKGs differ in terms of language, social and educational background, culture and heritage to name but a few factors. Such differences are not currently represented within the UKMED data.

Our data, and hence our findings, are specific to the UK context. Differential attainment/the awarding gap in the UK is associated with a range of variables; that include the characteristics which are the focus of this paper. These variables are likely to differ in different countries and are linked to societal and educational inequalities. Context may influence the variables themselves (e.g. minoritised groups), the extent of the DA associated with each variable, and how they intersect.

Finally, we appreciate that some of the terms used in this paper may not be preferred by all, and/or may not reflect the identities or lived experience of individuals and are likely to change over time.

### Implications for policy, practice and future research

Post-graduate medical examinations vary significantly in format, number of components, and delivery. More granular analyses are needed to ascertain whether these group-level differences exist in each postgraduate medical examination, the majority or a select few, and whether there are specialty-specific differences. Our results highlight the importance of considering all protected characteristics and examination formats when investigating DA in medical assessments.

Further research is also needed to see if the same patterns of performance are apparent in the high proportion of candidates who were not successful on the first sitting and go on to resit UK postgraduate examinations as well as those attempting more than one examination.

Quantitative studies such as this provide information on the “what” but not the “why” or “how” a gap in performance exists between different groups of doctors. In other words, they do not explore and identify whether a corresponding gap exists in learners’ experiences in the workplace, which might be contributing to DA. Qualitative studies are emerging in this area and these, combined with further quantitative work, are needed to uncover causes for protective factors against, DA (e.g. supportive relationships and work structures) and possible interventions to address DA (e.g. reverse mentoring, organisational-level change and interventions, including ensuring all groups are treated the same and, at an assessment level, ensuring assessment items are not biased against certain groups) [3, 52, 73, 77–80]. The findings from such studies can then be used to ensure equity across different groups in respect of educational and assessment processes. There is also the need to shift from studies which homogenise diverse groups (e.g. treating all IMGs as the same) to more nuanced studies that look at the outcomes and experiences of specific groups in more depth. Similarly, organisational contexts differ in their institutional structures and staff composition, and these local differences will influence colleagues’ experiences in the workplace, the type of interventions which may be appropriate and the effectiveness of any measures put in place to address differential attainment. Universal experience cannot be assumed, and thus, interventions and policies may need to be tailored to particular groups and places.

## Conclusions

This study of more than 180,000 examination attempts by UK (UKGs) and international medical graduates (IMGs) found statistically significant differences in performance on postgraduate medical examinations used in the UK according to place of primary qualification, gender, age, ethnicity, religion, sexual orientation, disability and LTFT status. These important findings warrant further, more granular analyses on an examination-by-examination basis to ascertain whether these group-level differences exist in each postgraduate medical examination, the majority or a select few. The findings from this study are important to examination candidates, medical educators, policymakers, those in charge of workforce planning and those with a legal duty to progress equity within medical education and training. Further research is needed to substantiate correlations and causality in relation to differences in group outcomes and the creation of more equitable workforce environments.

## Supplementary Information

Supplementary Material 1. Table 1. All UK postgraduate medical examinations included in the final analyses.

Supplementary Material 2. Table 2. Spearman’s Rho correlation coefficient matrix including all markers of socioeconomic status and educational background. All correlation coefficients demonstrated statistical significance P < 0.001.

Supplementary Material 3. Table 3. Intersectionality of Ethnicity and Religion variables. In total, 144,410 International and UK Medical Graduates had matched ethnicity and religion data, revealing a strong (Spearman’s Rho) correlation of 0.506 (p < 0.001). All values are given as percentages (total counts rounded to the nearest 5).

Supplementary Material 4. Table 4. Intersectionality of Ethnicity and Religion variables. In total, 104,205 UK Graduates had matched ethnicity and religion data, revealing a moderate (Spearman’s Rho) correlation of 0.396 (p < 0.001). All values are given as percentages (total counts rounded to the nearest 5).

## Data Availability

The dataset used in this study is collected by the GMC as part of its statutory duty to assure the quality of medical education and training. Data was deidentified by GMC staff before being provided to the researchers for analysis and interpretation of findings. Due to data protection regulations, the source data cannot be supplied. Anonymized summaries of it are supplied in this manuscript.

## Abbreviations

DA: Differential attainment
GMC: General Medical Council
IMD: Index of multiple deprivation
IMG: International (non-UK) medical graduate
LR: Logistic regression
LTFT: Less than full time
MV: Multi-variate
PLAB: Professional and Linguistic Assessments Board test
PMQ: Place of primary medical qualification (UK or international/non-UK graduate)
UCAS: Universities and Colleges Admissions Service
UK: United Kingdom
UKG: UK medical school graduate
UKMED: UK Medical Education Database
