# Supplementary material for: Differential attainment in UK postgraduate medical examinations: examining the relationship between sociodemographic differences and examination performance
Source: BMC Med. 2025 Apr 14;23:216. doi: 10.1186/s12916-025-04034-w (PMC11995605; doi:10.1186/s12916-025-04034-w)
Supplement: Supplementary file 1 — Supplementary Material 1. Table 1. All UK postgraduate medical examinations included in the final analyses. [file 12916_2025_4034_MOESM1_ESM.docx]

**Supplementary Table 1.** All UK postgraduate medical examinations included in the final analyses.

| **Examination title** |
| --- |
| Diploma in Otolaryngology – Head and Neck Surgery OSCE |
| Fellowship of the Faculty of Intensive Care Medicine MCQ |
| Fellowship of the Faculty of Intensive Care Medicine OSCE & Structured Oral Examination (SOE) |
| Final Fellowship of The Royal College of Radiologists (Part A) Clinical Oncology |
| Final Fellowship of The Royal College of Radiologists (Part A) Clinical Radiology |
| Final Fellowship of The Royal College of Radiologists (Part B) Clinical Oncology |
| Final Fellowship of The Royal College of Radiologists (Part B) Clinical Radiology |
| First Fellowship of The Royal College of Radiologists Clinical Oncology |
| First Fellowship of The Royal College of Radiologists Clinical Radiology |
| Fellowship of the Royal College of Anaesthetists Final SOE |
| Fellowship of the Royal College of Anaesthetists Final Written |
| Fellowship of the Royal College of Anaesthetists Primary MCQ |
| Fellowship of the Royal College of Anaesthetists Primary OSCE & SOE |
| Fellowship of the Royal College of Emergency Medicine OSCE |
| Fellowship of the Royal College of Emergency Medicine Written Components |
| Fellowship Exam of the Royal College of Ophthalmologists Part 1 |
| Fellowship Exam of the Royal College of Ophthalmologists Part 2 Oral |
| Fellowship Exam of the Royal College of Ophthalmologists Part 2 Written |
| Fellowship of the Royal College of Pathologists Part 1 |
| Fellowship of the Royal College of Pathologists Part 2 |
| Fellowship of the Royal Colleges of Surgeons Part 1 |
| Fellowship of the Royal Colleges of Surgeons Part 2 |
| Membership of Royal College of Emergency Medicine OSCE |
| Membership of Royal College of Emergency Medicine Written Components |
| Membership of Royal College of General Practice Applied Knowledge Test |
| Membership of Royal College of General Practice Clinical Skills Assessment |
| Membership of Royal College of Obstetrics & Gynaecology Part 3 |
| Membership of Royal College of Obstetrics & Gynaecology Parts 1 and 2 |
| Membership of the Royal College of Physicians Paces |
| Membership of the Royal College of Physicians Part 1 |
| Membership of the Royal College of Physicians Part 2 |
| Membership of the Royal College of Paediatrics and Child Health |
| Membership of the Royal College of Paediatrics and Child Health Clinical |
| Membership of the Royal Colleges of Surgeons Part A |
| Membership of the Royal Colleges of Surgeons Part B |
| Royal College of Ophthalmologists Refraction Certificate (Clinical) |
| Membership of the Royal College of Psychiatrists Clinical Assessment of Skills and Competencies |
| Membership of the Royal College of Psychiatrists Paper A |
| Membership of the Royal College of Psychiatrists Paper B |
| Specialty Certificate Examination (SCE) Acute Medicine (Written) |
| SCE Dermatology (Written) |
| SCE Endocrinology and Diabetes (Written) |
| SCE Gastroenterology (Written) |
| SCE Geriatric Medicine (Written) |
| SCE Nephrology (Written) |
| SCE Palliative Medicine (Written) |
| SCE Respiratory Medicine (Written) |
