# Supplementary material for: Differential attainment in UK postgraduate medical examinations: examining the relationship between sociodemographic differences and examination performance
Source: BMC Med. 2025 Apr 14;23:216. doi: 10.1186/s12916-025-04034-w (PMC11995605; doi:10.1186/s12916-025-04034-w)
Supplement: Supplementary file 2 — Supplementary Material 2. Table 2. Spearman’s Rho correlation coefficient matrix including all markers of socioeconomic status and educational background. All correlation coefficients demonstrated statistical significance P < 0.001. [file 12916_2025_4034_MOESM2_ESM.docx]

**Supplementary Table 2.** Spearman’s Rho correlation coefficient matrix including all markers of socioeconomic status and educational background. All correlation coefficients demonstrated statistical significance P<0.001.

| Parental Occupation | 1.00 |  |  |  |  |  |  |
| --- | --- | --- | --- | --- | --- | --- | --- |
| Parental Degree | -0.09 | 1.00 |  |  |  |  |  |
| Polar Quintile | -0.03 | 0.33 | 1.00 |  |  |  |  |
| IMD  Quintile | 0.06 | 0.29 | 0.74 | 1.00 |  |  |  |
| School Type | 0.05 | 0.26 | 0.53 | 0.52 | 1.00 |  |  |
| Income Support | -0.11 | 0.78 | 0.30 | 0.24 | 0.22 | 1.00 |  |
| Free School Meals | -0.12 | 0.82 | 0.31 | 0.26 | 0.24 | 0.87 | 1.00 |
|  | Parental Occupation | Parental Degree | Polar Quintile | IMD  Quintile | School Type | Income Support | Free School Meals |

|  | +1.00 |
| --- | --- |
|  |  |
|  |  |
|  |  |
|  | 0.00 |
|  |  |
|  |  |
|  |  |
|  | -1.00 |
