# Supplementary material for: Differential attainment in UK postgraduate medical examinations: examining the relationship between sociodemographic differences and examination performance
Source: BMC Med. 2025 Apr 14;23:216. doi: 10.1186/s12916-025-04034-w (PMC11995605; doi:10.1186/s12916-025-04034-w)
Supplement: Supplementary file 4 — Supplementary Material 4. Table 4. Intersectionality of Ethnicity and Religion variables. In total, 104,205 UK Graduates had matched ethnicity and religion data, revealing a moderate (Spearman’s Rho) correlation of 0.396 (p < 0.001). All values are given as percentages (total counts rounded to the nearest 5). [file 12916_2025_4034_MOESM4_ESM.docx]

**Supplementary Table 4.** Intersectionality of Ethnicity and Religion variables. In total, 104,205 UK Graduates had matched ethnicity and religion data, revealing a moderate (Spearman’s Rho) correlation of 0.396 (p<0.001). All values are given as percentages (total counts rounded to the nearest 5).

|  | No Religion | Buddhist | Christian | Hindu | Jewish | Muslim | Other | Sikh | Total |
| --- | --- | --- | --- | --- | --- | --- | --- | --- | --- |
| White | 56.4%  (39710) | <1%  (190) | 40.1%  (28785) | <1%  (5) | 1.2%  (825) | <1%  (275) | 1.0%  (670) | 0%  (0) | 70455 |
| Asian or Asian British | 22.0%  (5360) | 6.1%  (1485) | 12.7%  (3095) | 25.5%  (6190) | <1%  (5) | 25.3%  (6140) | 1.8%  (440) | 6.6%  (1605) | 24315 |
| Black or Black British | 8.8%  (210) | <1%  (5) | 77.1%  (1850) | <1%  (5) | <1%  (5) | 12.5%  (300) | 1.3%  (30) | 0%  (0) | 2400 |
| Mixed | 56.2% (2190) | 1.5%  (60) | 27.6%  (1075) | 1.3%  (50) | <1%  (25) | 9.7%  (380) | 2.8%  (110) | <1%  (10) | 3900 |
| Other | 25.4%  (795) | 5.4%  (170) | 24.9%  (780) | 1.6%  (50) | 1.0%  (30) | 37.6%  (1180) | 3.8%  (120) | <1%  (10) | 3135 |
